# Dibromidobis[1-(2,4,6-trimethyl­phen­yl)-1,4,5,6-tetra­hydro­pyrimidine-κ*N* ^3^]palladium(II)

**DOI:** 10.1107/S1600536810050968

**Published:** 2010-12-11

**Authors:** Pu Mao, Xiujun Liu, Liangru Yang, Jinwei Yuan, Maoping Song

**Affiliations:** a Department of Chemistry, Zhengzhou University, Zhengzhou 450001, People’s Republic of China; b School of Chemistry and Chemical Engineering, Henan University of Technology, Zhengzhou 450001, People’s Republic of China

## Abstract

In the title complex, [PdBr_2_(C_13_H_18_N_2_)_2_], the Pd^II^ atom is situated on an inversion center. The tetra­hydro­pyrimidine group of the *N*-(2,4,6-trimethyl­phen­yl)-1,4,5,6-tetra­hydro­pyrimidine ligand is twisted from the square (PdN_2_Br_2_) coordination plane with a C—N—Pd—Br torsion angle of 81.8 (4)°; this is different from the angle of 43.47 (14)°, reported in a closely related structure, dichloridobis(1-methyl-1,4,5,6-tetra­hydro­pyrimidine)­palladium(II).

## Related literature

For the related structure, dichloro­bis­(1-methyl-1,4,5,6-tetra­hydro­pyrimidine)­palladium(II), see: Chang & Lee (2007 ▶).
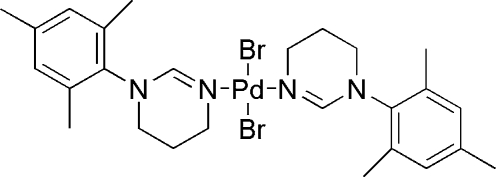


## Experimental

### 

#### Crystal data

[PdBr_2_(C_13_H_18_N_2_)_2_]
*M* *_r_* = 670.81
Monoclinic, 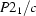

*a* = 7.1348 (14) Å
*b* = 21.308 (4) Å
*c* = 8.9704 (18) Å
β = 94.60 (3)°
*V* = 1359.4 (5) Å^3^
*Z* = 2
Mo *K*α radiation
μ = 3.64 mm^−1^
*T* = 293 K
0.20 × 0.20 × 0.20 mm

#### Data collection

Rigaku Saturn 724 CCD area-detector diffractometer
Absorption correction: multi-scan (*CrystalClear*; Rigaku/MSC, 2006 ▶) *T*_min_ = 0.530, *T*_max_ = 0.530
6809 measured reflections
2393 independent reflections
1931 reflections with *I* > 2σ(*I*)
*R*_int_ = 0.034

#### Refinement

*R*[*F* ^2^ > 2σ(*F* ^2^)] = 0.045
*wR*(*F* ^2^) = 0.114
*S* = 1.08
2393 reflections
154 parameters
H-atom parameters constrained
Δρ_max_ = 0.76 e Å^−3^
Δρ_min_ = −0.50 e Å^−3^

### 

Data collection: *CrystalClear* (Rigaku/MSC, 2006 ▶); cell refinement: *CrystalClear*; data reduction: *CrystalClear*; program(s) used to solve structure: *SHELXS97* (Sheldrick, 2008 ▶); program(s) used to refine structure: *SHELXL97* (Sheldrick, 2008 ▶); molecular graphics: *SHELXTL* (Sheldrick, 2008 ▶); software used to prepare material for publication: *SHELXL97*.

## Supplementary Material

Crystal structure: contains datablocks I, global. DOI: 10.1107/S1600536810050968/su2226sup1.cif

Structure factors: contains datablocks I. DOI: 10.1107/S1600536810050968/su2226Isup2.hkl

Additional supplementary materials:  crystallographic information; 3D view; checkCIF report

## Figures and Tables

**Fig. 1.**
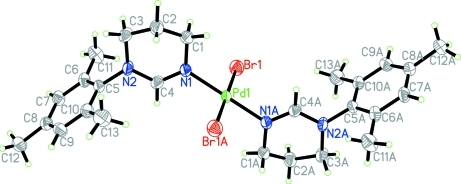
The molecular structure of the title molecule, showing 30% probability displacement ellipsoids.

## supplementary crystallographic information

### Comment

Our group is interested in the preparation of new N-heterocyclic carbene (NHC) ligands based on substituted 1,4,5,6-tetrahydropyrimidine and their palladium complexes. In the course of preparing the palladium complex of a bidentate NHC ligand, we observed that the reaction of the corresponding tetrahydropyrimidine salt and Pd(OAc)_2_, unexpectedly, under unoptimized reaction conditions, afforded the title compound.

In the title compound the Pd atom is situated on a center of inversion (Fig. 1). The organic ligands twist away from the square (PdN2Br2) coordination plane with a C1—N1—Pd1—Br1 torsion angle of 81.8 (4)°. The corresponding angle in the closely related structure, dichlorobis(1-methyl-1,4,5,6-tetrahydropyrimidine)palladium(II) [Chang & Lee, 2007], is 43.47 (14)°.

### Experimental

Pd(OAc)_2_ (101 mg, 0.45 mmol) was added to a solution of N,N-methylene-N',N'-bis-2,4,6-trimethylphenyl-1,4,5,6-tetrahydropyrimidine (260 mg, 0.45 mmol) in DMSO (3 ml). The mixture was then heated at 333 K for 5 h. After cooling, the solvent was removed completely under vacuum. The residue was then dissolved in CHCl_3_, and filtered. Evaporation of the filtrate afforded an orange solid (yield 200 mg, 66%). Crystals of the title complex, suitable for structural analysis, were obtained by vapor diffusion of diethyl ether into an acetonitrile solution containing the solid.

### Refinement

The hydrogen atoms were included in calculated positions and treated as riding: C-H = 0.93, 0.97 and 0.96 Å, for CH, CH_2_ and CH_3_ H-atoms, respectively, with U_iso_(H) = k × U_eq_(C), where k = 1.5 for CH_3_ H-atoms and k = 1.2 for all other H-atoms.

### Crystal data

**Table:**

[PdBr_2_(C_13_H_18_N_2_)_2_]	*F*(000) = 672
*M**_r_* = 670.81	*D*_x_ = 1.639 Mg m^−^^3^
Monoclinic, *P*2_1_/*c*	Mo *K*α radiation, λ = 0.71073 Å
Hall symbol: -P 2ybc	Cell parameters from 3263 reflections
*a* = 7.1348 (14) Å	θ = 2.5–27.9°
*b* = 21.308 (4) Å	µ = 3.64 mm^−^^1^
*c* = 8.9704 (18) Å	*T* = 293 K
β = 94.60 (3)°	Prismatic, colourless
*V* = 1359.4 (5) Å^3^	0.20 × 0.20 × 0.20 mm
*Z* = 2	

### Data collection

**Table:**

Rigaku CCD area-detector diffractometer	2393 independent reflections
Radiation source: fine-focus sealed tube	1931 reflections with *I* > 2σ(*I*)
graphite	*R*_int_ = 0.034
ω scans	θ_max_ = 25.0°, θ_min_ = 2.5°
Absorption correction: multi-scan (*CrystalClear*; Rigaku/MSC, 2006)	*h* = −8→5
*T*_min_ = 0.530, *T*_max_ = 0.530	*k* = −18→25
6809 measured reflections	*l* = −9→10

### Refinement

**Table:**

Refinement on *F*^2^	Primary atom site location: structure-invariant direct methods
Least-squares matrix: full	Secondary atom site location: difference Fourier map
*R*[*F*^2^ > 2σ(*F*^2^)] = 0.045	Hydrogen site location: inferred from neighbouring sites
*wR*(*F*^2^) = 0.114	H-atom parameters constrained
*S* = 1.08	*w* = 1/[σ^2^(*F*_o_^2^) + (0.0553*P*)^2^] where *P* = (*F*_o_^2^ + 2*F*_c_^2^)/3
2393 reflections	(Δ/σ)_max_ < 0.001
154 parameters	Δρ_max_ = 0.76 e Å^−^^3^
0 restraints	Δρ_min_ = −0.50 e Å^−^^3^

### Special details

**Table:**

Geometry. All e.s.d.'s (except the e.s.d. in the dihedral angle between two l.s. planes) are estimated using the full covariance matrix. The cell e.s.d.'s are taken into account individually in the estimation of e.s.d.'s in distances, angles and torsion angles; correlations between e.s.d.'s in cell parameters are only used when they are defined by crystal symmetry. An approximate (isotropic) treatment of cell e.s.d.'s is used for estimating e.s.d.'s involving l.s. planes.
Refinement. Refinement of *F*^2^ against ALL reflections. The weighted *R*-factor *wR* and goodness of fit *S* are based on *F*^2^, conventional *R*-factors *R* are based on *F*, with *F* set to zero for negative *F*^2^. The threshold expression of *F*^2^ > σ(*F*^2^) is used only for calculating *R*-factors(gt) *etc*. and is not relevant to the choice of reflections for refinement. *R*-factors based on *F*^2^ are statistically about twice as large as those based on *F*, and *R*- factors based on ALL data will be even larger.

### Fractional atomic coordinates and isotropic or equivalent isotropic displacement parameters (Å^2^)

**Table:**

	*x*	*y*	*z*	*U*_iso_*/*U*_eq_	
Pd1	0.5000	0.5000	0.0000	0.0445 (2)	
Br1	0.27385 (9)	0.45987 (3)	−0.19525 (7)	0.0700 (2)	
N1	0.3804 (6)	0.44800 (17)	0.1532 (5)	0.0502 (11)	
N2	0.4027 (6)	0.36304 (16)	0.3218 (5)	0.0513 (11)	
C1	0.2139 (8)	0.4701 (2)	0.2167 (6)	0.0595 (16)	
H1A	0.1044	0.4545	0.1570	0.071*	
H1B	0.2111	0.5156	0.2118	0.071*	
C2	0.2026 (10)	0.4506 (2)	0.3718 (7)	0.0722 (19)	
H2A	0.0783	0.4607	0.4015	0.087*	
H2B	0.2929	0.4747	0.4348	0.087*	
C3	0.2385 (8)	0.3831 (2)	0.3997 (7)	0.0659 (17)	
H3A	0.2622	0.3757	0.5062	0.079*	
H3B	0.1290	0.3589	0.3638	0.079*	
C4	0.4604 (8)	0.3983 (2)	0.2102 (6)	0.0534 (14)	
H4	0.5700	0.3855	0.1695	0.064*	
C5	0.5155 (7)	0.3102 (2)	0.3749 (6)	0.0461 (12)	
C6	0.4605 (8)	0.2496 (2)	0.3291 (6)	0.0541 (14)	
C7	0.5759 (8)	0.1998 (2)	0.3757 (6)	0.0586 (15)	
H7	0.5389	0.1592	0.3491	0.070*	
C8	0.7456 (8)	0.2088 (3)	0.4614 (7)	0.0631 (16)	
C9	0.7929 (8)	0.2699 (2)	0.5081 (7)	0.0628 (15)	
H9	0.9027	0.2766	0.5691	0.075*	
C10	0.6784 (8)	0.3208 (2)	0.4650 (7)	0.0561 (14)	
C11	0.2792 (9)	0.2376 (3)	0.2354 (7)	0.0757 (18)	
H11A	0.1756	0.2527	0.2871	0.114*	
H11B	0.2649	0.1933	0.2180	0.114*	
H11C	0.2817	0.2590	0.1415	0.114*	
C12	0.8751 (9)	0.1538 (3)	0.5026 (9)	0.087 (2)	
H12A	0.9802	0.1548	0.4423	0.130*	
H12B	0.8074	0.1152	0.4855	0.130*	
H12C	0.9194	0.1567	0.6063	0.130*	
C13	0.7335 (9)	0.3853 (3)	0.5220 (8)	0.081 (2)	
H13A	0.7528	0.4123	0.4390	0.122*	
H13B	0.8477	0.3826	0.5861	0.122*	
H13C	0.6352	0.4021	0.5773	0.122*	

### Atomic displacement parameters (Å^2^)

**Table:**

	*U*^11^	*U*^22^	*U*^33^	*U*^12^	*U*^13^	*U*^23^
Pd1	0.0507 (4)	0.0410 (3)	0.0427 (4)	0.0089 (2)	0.0098 (3)	0.0122 (2)
Br1	0.0733 (5)	0.0738 (4)	0.0615 (5)	−0.0017 (3)	−0.0030 (3)	0.0054 (3)
N1	0.054 (3)	0.045 (2)	0.053 (3)	0.0112 (19)	0.012 (2)	0.0117 (19)
N2	0.066 (3)	0.038 (2)	0.052 (3)	0.0043 (19)	0.016 (2)	0.0131 (18)
C1	0.060 (4)	0.046 (3)	0.075 (4)	0.014 (2)	0.023 (3)	0.010 (3)
C2	0.084 (5)	0.061 (3)	0.076 (5)	0.027 (3)	0.034 (4)	0.016 (3)
C3	0.078 (4)	0.055 (3)	0.070 (4)	0.013 (3)	0.040 (4)	0.011 (3)
C4	0.056 (4)	0.052 (3)	0.054 (3)	0.005 (2)	0.020 (3)	0.005 (2)
C5	0.048 (3)	0.041 (3)	0.049 (3)	0.008 (2)	0.006 (2)	0.010 (2)
C6	0.068 (4)	0.042 (3)	0.053 (3)	0.004 (2)	0.005 (3)	0.004 (2)
C7	0.071 (4)	0.036 (3)	0.069 (4)	0.004 (2)	0.006 (3)	0.004 (2)
C8	0.058 (4)	0.056 (3)	0.077 (4)	0.011 (3)	0.017 (3)	0.019 (3)
C9	0.049 (4)	0.061 (3)	0.077 (4)	0.002 (3)	−0.006 (3)	0.011 (3)
C10	0.057 (4)	0.039 (3)	0.073 (4)	−0.002 (2)	0.005 (3)	0.006 (2)
C11	0.085 (5)	0.057 (3)	0.081 (5)	0.006 (3)	−0.017 (4)	−0.016 (3)
C12	0.070 (5)	0.070 (4)	0.121 (6)	0.023 (3)	0.014 (4)	0.031 (4)
C13	0.073 (4)	0.054 (3)	0.115 (6)	−0.012 (3)	−0.009 (4)	−0.006 (3)

### Geometric parameters (Å, °)

**Table:**

Pd1—N1^i^	2.008 (4)	C5—C6	1.402 (7)
Pd1—N1	2.008 (4)	C6—C7	1.386 (7)
Pd1—Br1^i^	2.4391 (9)	C6—C11	1.507 (8)
Pd1—Br1	2.4391 (9)	C7—C8	1.394 (8)
N1—C4	1.289 (6)	C7—H7	0.9300
N1—C1	1.437 (6)	C8—C9	1.401 (8)
N2—C4	1.343 (6)	C8—C12	1.519 (7)
N2—C5	1.442 (6)	C9—C10	1.394 (7)
N2—C3	1.474 (6)	C9—H9	0.9300
C1—C2	1.461 (8)	C10—C13	1.508 (7)
C1—H1A	0.9700	C11—H11A	0.9600
C1—H1B	0.9700	C11—H11B	0.9600
C2—C3	1.478 (7)	C11—H11C	0.9600
C2—H2A	0.9700	C12—H12A	0.9600
C2—H2B	0.9700	C12—H12B	0.9600
C3—H3A	0.9700	C12—H12C	0.9600
C3—H3B	0.9700	C13—H13A	0.9600
C4—H4	0.9300	C13—H13B	0.9600
C5—C10	1.380 (7)	C13—H13C	0.9600
			
N1^i^—Pd1—N1	180.000 (1)	C6—C5—N2	119.1 (5)
N1^i^—Pd1—Br1^i^	90.30 (14)	C7—C6—C5	118.1 (5)
N1—Pd1—Br1^i^	89.70 (14)	C7—C6—C11	120.0 (5)
N1^i^—Pd1—Br1	89.70 (13)	C5—C6—C11	121.8 (5)
N1—Pd1—Br1	90.30 (14)	C6—C7—C8	122.0 (5)
Br1^i^—Pd1—Br1	180.0	C6—C7—H7	119.0
C4—N1—C1	117.8 (4)	C8—C7—H7	119.0
C4—N1—Pd1	121.7 (3)	C7—C8—C9	118.0 (5)
C1—N1—Pd1	120.0 (3)	C7—C8—C12	120.9 (5)
C4—N2—C5	119.1 (4)	C9—C8—C12	121.1 (6)
C4—N2—C3	119.6 (4)	C10—C9—C8	121.2 (6)
C5—N2—C3	120.9 (4)	C10—C9—H9	119.4
N1—C1—C2	113.2 (4)	C8—C9—H9	119.4
N1—C1—H1A	108.9	C5—C10—C9	118.9 (4)
C2—C1—H1A	108.9	C5—C10—C13	122.1 (5)
N1—C1—H1B	108.9	C9—C10—C13	118.9 (5)
C2—C1—H1B	108.9	C6—C11—H11A	109.5
H1A—C1—H1B	107.7	C6—C11—H11B	109.5
C1—C2—C3	114.5 (5)	H11A—C11—H11B	109.5
C1—C2—H2A	108.6	C6—C11—H11C	109.5
C3—C2—H2A	108.6	H11A—C11—H11C	109.5
C1—C2—H2B	108.6	H11B—C11—H11C	109.5
C3—C2—H2B	108.6	C8—C12—H12A	109.5
H2A—C2—H2B	107.6	C8—C12—H12B	109.5
N2—C3—C2	109.6 (4)	H12A—C12—H12B	109.5
N2—C3—H3A	109.7	C8—C12—H12C	109.5
C2—C3—H3A	109.7	H12A—C12—H12C	109.5
N2—C3—H3B	109.7	H12B—C12—H12C	109.5
C2—C3—H3B	109.7	C10—C13—H13A	109.5
H3A—C3—H3B	108.2	C10—C13—H13B	109.5
N1—C4—N2	127.1 (5)	H13A—C13—H13B	109.5
N1—C4—H4	116.5	C10—C13—H13C	109.5
N2—C4—H4	116.5	H13A—C13—H13C	109.5
C10—C5—C6	121.6 (4)	H13B—C13—H13C	109.5
C10—C5—N2	119.2 (4)		
			
Br1^i^—Pd1—N1—C4	−73.6 (4)	C3—N2—C5—C6	85.6 (7)
Br1—Pd1—N1—C4	106.4 (4)	C10—C5—C6—C7	−0.7 (8)
Br1^i^—Pd1—N1—C1	98.2 (4)	N2—C5—C6—C7	177.0 (4)
Br1—Pd1—N1—C1	−81.8 (4)	C10—C5—C6—C11	177.7 (5)
C4—N1—C1—C2	24.7 (8)	N2—C5—C6—C11	−4.7 (8)
Pd1—N1—C1—C2	−147.4 (4)	C5—C6—C7—C8	−2.2 (8)
N1—C1—C2—C3	−48.5 (8)	C11—C6—C7—C8	179.4 (5)
C4—N2—C3—C2	−18.5 (8)	C6—C7—C8—C9	3.9 (8)
C5—N2—C3—C2	154.1 (5)	C6—C7—C8—C12	−176.1 (5)
C1—C2—C3—N2	44.1 (8)	C7—C8—C9—C10	−2.9 (9)
C1—N1—C4—N2	1.8 (9)	C12—C8—C9—C10	177.2 (5)
Pd1—N1—C4—N2	173.8 (4)	C6—C5—C10—C9	1.7 (8)
C5—N2—C4—N1	−177.5 (5)	N2—C5—C10—C9	−176.0 (5)
C3—N2—C4—N1	−4.7 (9)	C6—C5—C10—C13	−176.5 (5)
C4—N2—C5—C10	75.9 (7)	N2—C5—C10—C13	5.8 (8)
C3—N2—C5—C10	−96.7 (6)	C8—C9—C10—C5	0.1 (8)
C4—N2—C5—C6	−101.8 (6)	C8—C9—C10—C13	178.4 (6)

Symmetry codes: (i) −*x*+1, −*y*+1, −*z*.
